# The Effect of Nanosilver Sodium Fluoride on the Mechanical and Physiochemical Properties of Artificially Demineralised Dentin

**DOI:** 10.3290/j.ohpd.b4116081

**Published:** 2023-05-24

**Authors:** Syed Saad Bin Qasim, Jagan Kumar Baskaradoss, Ahmed Meslam Mohamed, Colin Alexandar Murray, Umer Daood, Mirza Rustum Baig

**Affiliations:** a Assistant Professor, Department of Bioclinical Sciences, Faculty of Dentistry, Kuwait University, Kuwait City, Kuwait. Conceived the idea, conducted the experimental work, interpreted the data, wrote and edited the final manuscript.; b Assistant Professor, Department of Developmental and Preventive Sciences, Kuwait University, Kuwait City, Kuwait. Conceived the idea, performed the statistical analysis, reviewed, edited, and approved the final manuscript.; c Associate Researcher, Department of Chemistry, Faculty of Science, Kuwait University, Kuwait City, Kuwait. Experimental analysis, wrote the manuscript, interpreted the data.; d Professor, Department of Preventive and Restorative Dentistry, College of Dental Medicine, University of Sharjah, Sharjah, UAE, Wrote, reviewed, and edited the final manuscript.; e Associate Professor, Restorative Division, Faculty of Dentistry, International Medical University, Kuala Lumpur, Malaysia. Wrote, reviewed, and edited the final manuscript.; f Associate Professor, Department of Restorative Sciences, Faculty of Dentistry, Kuwait University, Kuwait City, Kuwait. Interpreted the data, wrote, edited, and reviewed the final version of the manuscript.

**Keywords:** caries, demineralisation, fluoride, nanoparticles, root dentin, silver

## Abstract

**Purpose:**

To synthesise and characterise nanosilver sodium fluoride (NSSF) and assess the effect of applying this formulation in vitro on artificially demineralised root dentin lesions, compared with the application of silver diamine fluoride (SDF), sodium fluoride (NAF) or no treatment, in terms of mechanical, chemical and ultrastructural properties.

**Materials and Methods:**

NSSF was prepared using 0.5 wt% chitosan solution. On 40 extracted human molars, the buccal aspect of the cervical thirds of roots were prepared and divided into 4 groups of 10 each: control (no treatment), NSSF, SDF and NaF (n = 10). The specimens were examined using scanning electron microscopy (SEM), atomic force microscopy (AFM), and x-ray photoelectron spectroscopy (XPS). Fourier transform infrared spectroscopy (FTIR), surface and cross-sectional microhardness and nano-indentation tests were performed to determine the mineral and carbonate content, microhardness, and nanohardness, respectively. Statistical analysis was performed to determine the differences between the different treatment groups for the set parameters using parametric and non-parametric tests. Tukey’s and Dunnet’s T3 post-hoc tests were further used for multiple comparisons between groups (α = 0.05).

**Results:**

The control group (no treatment) was found to have statistically significantly lower mean scores for surface and cross-sectional microhardness compared with all other test groups (NaF, NSSF and SDF) (p < 0.05). Spearman’s rank correlation test showed statistically insignificant differences between the mineral-to-matrix ratio (M:M) and carbonate content of all groups (p < 0.05).

**Conclusions:**

Treatment of root lesions with NSSF yielded comparable results to SDF and NaF under in-vitro conditions.

There are multiple oral and systemic challenges facing the geriatric population, whose disability and frailty may lead to the increased possibility of developing root caries (RC) on exposed root surfaces due to gingival recession, plaque retention and hyposalivation.^1^ Furthermore, co-morbidities – such as medications related to xerostomia, less exposure to fluoride, frequency of carbohydrate intake and limited manual dexterity for plaque control – accumulate and thereby exaggerate the occurrence of RC lesions.^22^ Such carious lesions can either be cavitated or non-cavitated and are usually located below the cementoenamel junction (CEJ). It may present as a discoloured, ill-defined, softened lesion mostly involving the dentin and cementum.^1^ A few systematic reviews and meta-analyses which analysed the occurrence of RC^14,20^ have reported annual RC incidence rates at 18.25% (CI = 13.22%–23.28%).^14^

Therapeutic strategies that focus on a non-invasive approach for treating root caries defects include general advice from the clinician to improve tooth brushing with a fluoridated toothpaste^20^ or application of other agents as adjuncts to fluoride (F^-^), such as chlorhexidine, amorphous calcium phosphate and silver diamine fluoride (SDF).^1^ The clinical efficacy of currently recommended preventive strategies (22600 ppm fluoride varnish,^13^ or 38% SDF with 44800 ppm) infer that high concentrations of fluorides might be effective in tackling RC.^37^ However, to the authors’ best knowledge, the scientific literature currently contains no studies demonstrating the direct effect of fluoride hindering the breakdown of collagen fibril network and causing deactivation of endogenous proteases.^4^

SDF is a colourless ammonia solution made with silver (Ag) and fluoride (F^-^) ions in concentrations that are more than sufficient for remineralisation of dental hard tissues.^23^ A common drawback to SDF use reported in the literature is black discolouration of tooth surface after application of the SDF solution due to excess free Ag^+^ ions precipitating on the tooth surface.^6,12^ The blackening effect occurs within 2 min of application and the degree of the stain intensifies in the 6 h following application.^6^ Manufacturers identified this shortcoming and modified the formulation by adding potassium iodide, making it a two-stage intervention relying on a first application of Ag solution and then a separate application of potassium iodide.^13,32^ Potassium iodide is a salt, forming white hexahedral crystals, which purportedly reduce the anti-caries effect of SDF by decreasing the number of available Ag+ ions.^6^ Potassium iodide has been shown to cause a metallic taste in the mouth along with swollen glands, nausea, diarrhoea and vomiting.^12^ More recently, the use of nanosilver sodium fluoride (NSSF) has also been reported.^11,38^ Different formulations with varying concentrations of Ag, F and chitosan have been investigated and the effect of nanoparticles needs more in-depth examination to explore the benefits. Ag nanoparticles have been used in several dental^24^ and biomedical applications, such as drug delivery, cancer therapy and as antimicrobials when used in low concentrations.^38^ Due to their inherent optoelectronic properties, nano-sized Ag particles result in less discolouration. Other important parameters that might influence the properties of such nanoparticles are ion release, surface area, surface charge, concentration and colloidal state.^41^

Therefore, the aim of the current investigation was to synthesise nanosilver sodium fluoride (NSSF) in vitro and analyse the solution to assess its effect on artificially demineralised root dentin, in comparison with commercially available SDF, sodium fluoride (NaF), and no treatment (control), in terms of mechanical, chemical and ultrastructural properties. The null hypothesis was that there would be no differences between control, NSSF, SDF, and NaF, when used on the artificially demineralised root dentin in therms of microhardness, nanohardness, chemical profile, and mineral content.

## Materials and Methods

**Fig 1:**
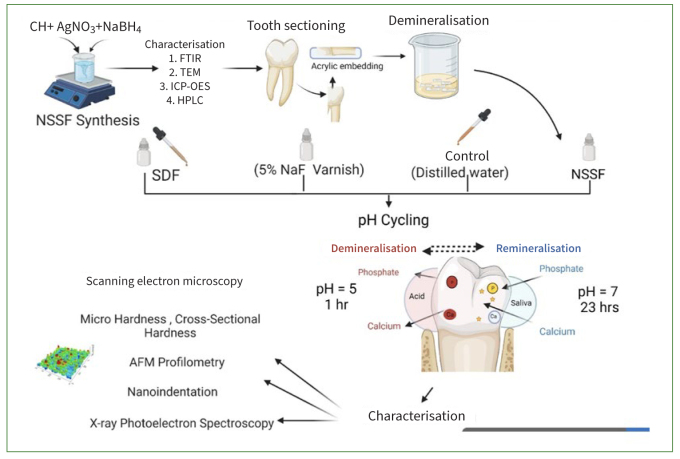
Schematic illustration of the experimental treatment procedures.

Chitosan (CH) (ChitoClear; Siglufjörður, Iceland) with a molecular weight of 133 kDa (degree of deacetylation = 96.6%), glacial acetic acid, sodium borohydride (NaBH_4_) and sodium hydroxide (NaOH) (Sigma Aldrich; St Louis, MO, USA), silver nitrate (Riedel-deHaën; Hannover, Germany) and sodium fluoride (NaF) (Merck; Darmstadt, Germany) were used in this study for synthesising the different formulations tested on the teeth. The schematic diagram of the experiments performed are shown in Fig 1. Table 1 shows the details of the biomaterials used for treating demineralised dentin.

**Table 1:** Details of the biomaterials used for treating demineralised dentin

Treatment	Commercial name	Active ingredients	Final state	Manufacturer
Silver diamine fluoride	Riva Star	30-35% silver fluoride (0.06 g/ml F-ions), 60% ammonia solution, saturated ammonia solutions	Liquid	SDI; Bayswater, Australia
Fluoride varnish	Enamelast	5% sodium fluoride	Varnish	Ultradent; South Jordan, UT, USA
Nanosilver sodium fluoride	N/A	Nanosilver particles, sodium fluoride	Liquid	In-vitro lab based
Distilled water	Control	N/A	Liquid	N/A

### Synthesis of Nanosilver sodium fluoride (NSSF)

The synthesis of nanosilver sodium fluoride was adapted from dos Santos et al,^8^ as follows: 0.3 g of CH and 58.8 ml of distilled water were mixed and stirred for 20 min. 1.2 ml of acetic acid (0.2 M) was then added dropwise to the solution and stirred for 3 h to prepare a 0.5 wt % solution. Once completely dissolved, the solution was placed in an ice bath and precooled. 0.012 M silver nitrate (4 ml) was added dropwise to the CH solution. After 15 min, sodium borohydride was added, maintaining the ratio of silver nitrate to sodium borohydride at 1:6 (0.072 M). The reaction was undertaken in the dark and once the colour change was noticed, sodium fluoride (300 mg) at a concentration of 5028 µg/ml was added to stabilise the reaction.

### Characterisation of NSSF

The synthesised NSSF was characterised using UV-vis spectroscopy (Eppendorf, Biospectrometer basic; Hamburg, Germany) by running a blank spectrum of neat CH solution as a background. Spectral data were obtained at wavelengths from 200 to 500 nm. 10 µl of solution was placed on a copper grid and allowed to air dry in a fume hood for 20 min, followed by visualisation using transmission electron microscopy (TEM) (JEM, 1200 EX11; Tokyo, Japan) at an accelerating voltage of 100 kV. Fourier transform infrared spectroscopy (FTIR, Tensor 27 system, Bruker Optics; Ettlingen, Germany) was performed by placing a droplet of the solution onto the attenuated total reflectance (ATR) crystal, and spectra were acquired immediately afterwards. The spectral range was from 400 to 4000 cm^-1^. A total of 32 scans were conducted at a resolution of 4 cm^-1^. Data were exported into CSV and then replotted in OMNIC Software (Thermo Fisher Scientific; Waltham, MA, USA). Particle size analysis was conducted using a Zetasizer (Zetasizer Nano Series, Malvern Instruments; Malvern, Worcestershire, UK). All measurements were conducted in triplicate with a temperature equilibrium of 1 min at 25°C. The instrument was equipped with a standard red helium-neon laser, set at 4.0 mW and 632.8 nm. The measurement was conducted at a fixed scattering anlge of 173 degrees. The data processing was set at high multi-modal resolution.

### Sample Preparation

Human molars (n = 90) extracted for periodontal reasons were collected in accordance with the protocol approved by the University Health Science Centre Ethics Committee, in full accordance with the World Medical Association Declaration of Helsinki (approval number 6100). The teeth were stored in 0.1% thymol solution at 4°C until use and then cleaned manually with hand scalers and curettes before specimen preparation. The crown and apical root portions were sectioned using a water-cooled slow-speed diamond saw (Accutom 100, Struers; Ballerup, Denmark) and the specimens were embedded in epoxy resin (Epofix kit, Struers). Exposed surfaces were smoothed using 600-, 800-, 1200- and 2000-grit silicon carbide disks (CarbiMet, Buehler; Lake Bluff, IL, USA) and then polished with 3-µm diamond polishing paste (MetaDi polycrystalline diamond suspension, Buehler). The buccal aspect of the roots adjacent to the cementoenamel junction (Fig 1) was prepared leaving a small window of 4 x 4 mm on each specimen for applying different treatments, while the remaining portions were covered with nail varnish. All the specimens were prepared for surface and cross-sectional microhardness analysis followed by surface nano-indentation and atomic force microscopy (AFM). Specimens used for micro- and nano-hardness were further analysed with FTIR and x-ray photoelectron spectroscopy (XPS) to calculate the mineral:matrix ratio and carbonate content. Three specimens were randomly chosen from each of the four test groups for analysis using scanning electron microscopy (SEM) and EDX. The minimum sample size was estimated to achieve a power of 80% with a confidence level of 95% using the G-Power version 3.1 programme. The effect size was calculated as 0.882, based on a previous study. A-priori one-way ANOVA determined that a minimum of 6 samples in each group were required for testing cross-sectional microhardness for the 3 groups. This was then rounded up to 10 samples per group (N = 40).

### Demineralisation Protocol

The demineralisation gel was prepared by mixing 0.1 M NaOH with 0.1 M lactic acid to achieve a pH of 4.5. Then, 6% w/v hydroxyethyl cellulose (Sigma Aldrich) was added to the solution, stirred for 1 h and let stand for 24 h until a gel consistency was achieved.^26^ The mounted tooth samples were completely immersed in the gel to ensure coverage of all the prepared root surfaces. The specimens remained in the gel for 11 days at room temperature, and pH was checked regularly at 24-h intervals. At the end of the procedure, specimens were thoroughly cleaned under running distilled water and stored (4°C) until further use.

### Treatment Protocol

Block randomisation was used to assign the demineralised specimens to 4 groups (control, NSSF, SDF and NaF; n = 10). Control specimens were treated with distilled water. The experimental formulation was applied on the demineralised root dentin for 20 s and allowed to dry for 1 min. Excess solution was removed using a micro cotton pellet. The respective manufacturer’s recommendations were followed for the other commercial products (SDF and NaF). The surfaces of the specimens were dried and SDF or NaF were applied using a saturated microbrush for 10 s. Immediately thereafter, potassium iodide (KI) was applied used.^15^ Finally, all specimens were dried and stored in distilled water until further characterisation. More information about the materials used is provided in Table 1.

### pH Cycling

After finishing the treatment, all specimens underwent cyclic demineralisation and remineralisation (8 days).^18^ Demineralising solution was made with 50 mM acetic acid, 1.5 mM calcium chloride (CaCl_2_) and 0.9 mM potassium dihydrogen phosphate (KH_2_PO_4_), with the pH adjusted to 5 using potassium hydroxide (KOH). The remineralising solution was made using 1.5 mM CaCl_2_, 0.9 mM KH_2_PO_4_, 130 mM potassium chloride (KCl) and 20 mM HEPES buffer; the pH was adjusted to 7 using potassium hydroxide (KOH). Specimens were immersed in demineralisation solution for 1 h and remineralisation solution for 23 h at 37°C. After each consecutive cycle, specimens were washed and placed in distilled water.^3^ The aim of the pH cycling models was to mimic the dynamic environment of mineral loss and gain involved in caries formation.

### Surface Microhardness

Surface microhardness of the root dentin specimens was conducted using a Vickers diamond indenter (Innovatest; Maastricht, Netherlands). Ten indentations 50 µm apart were performed on each specimen of the control and test groups (SDF, NaF and NSSF). The tests were conducted using a digital microhardness tester with a load of 20 g for 20 s on the surface of the specimen (CV instruments 400DAT/ 3; Sheffield, UK) at room temperature.

### Cross-sectional Microhardness

After surface microhardness evaluation was completed, the specimens were longitudinally sectioned, re-embedded in acrylic resin, and polished using a water-cooled grinding and polishing unit with 200-, 600- and 1000-grit SiC abrasive papers (CarbiMet, Buehler) and 0.6-µm diamond suspension (MetaDi polycrystalline diamond suspension, Buehler). Debris on the specimens was removed by placing them in an ultrasonic bath containing distilled water. Cross-sectional microhardness testing was performed perpendicular to the demineralised surface at depths of 25 to 50 µm and 50 to 100 µm below the surface with a load of 20 g and dwell time of 20 s for each indentation. Microhardness in g/µm^2^ was calculated using the equation:

*HV* = 1.854 * *P / d2*

where HV is the Vickers Hardness, P is the load set in grams (g) and d is the diagonal’s length in µm.

### Nano-Indentation and Atomic Force Microscopy (AFM)

Nano-indentations of the surface were performed using a Hysitron TI 700 Ubi Triboindenter (Bruker-Hysitron; Minneapolis, MN, USA) equipped with a Tribosacan system (Bruker-Hysitron). A Berkovich tip (tip radius: 100 nm) was used. The instrument was calibrated according to the approach proposed by Oliver and Pharr in 1992.^2,15,26^ It was applied onto the specimens at a maximum force of 800 µN using a standard load-controlled test for each individual indentation that consisted of three segments: loading, holding at the peak, and unloading, in which each of the three segments lasted for 5 s. While the indentation was being performed, the applied load P and displacement h were continuously monitored and recorded, which aided in formulating a load-deformation curve. The hardness was calculated by dividing the projected contact area at maximum load, and the elastic modulus was calculated from the contact stiffness. Nanohardness values were reported in H (GPa) and reduced elastic modulus (or reduced Young’s modulus) E_r_ (GPa). A linear series of 10 indentations were conducted which were approximately 10 µm apart. The Nano-Observer CSI-AFM (atomic force microscope, Concept Scientific Instruments; Les Ulis, France) was used to visualise the topographical mapping of surface and cross-sectioned specimens. Images were acquired in the region of 50 µm x 50 µm at a scan rate of 0.75 ln/s. The images were acquired using a tapping mode with a calibrated, vertically engaged piezo scanner.

### Fourier Transform Infrared Spectroscopy

Fourier transform infrared (FTIR) spectroscopy was performed using the attenuated total reflectance mode (ATR) (Bruker, Tensor 27 system, Bruker Optics) equipped with a diamond ATR crystal. Spectra were collected from neat chitosan solution (CH), silver nitrate, sodium fluoride and synthesised NSSF in the mid-infrared region of 400 to 4000 cm^-1^ at a spectral resolution of 4 cm^-1^. A total of 32 scans per pixel were acquired. A background spectrum was collected prior to analysing the specimens to eliminate the atmospheric background, primarily water vapor and carbon dioxide. This background spectrum was taken every 30 min to ensure accurate background subtraction. The obtained spectra were processed using OMNIC 9 Software (Thermo Fisher Scientific).

After pH cycling, dentin specimens were scanned from 800 cm^-^^1^ to 2000 cm^-1^. A total of 20 spectra were collected from the root dentin area of the control, SDF, NaF and NSSF specimens. These were collected in OPUS software (version 6.5, Bruker; Billerica, MA, USA) and the data points then exported to OMNIC 9 software for further analysis. The determination of band area was performed after baseline correction using the peak area tool. The band ratio between absorbance of 1026 cm^-1^ and 1655 cm^-1^ (A1026 / A1655) which are assigned to ν3 PO_4_^3-^ of the hydroxyapatite phosphate ion and the C=O stretching of collagen amide I, respectively, were analysed to represent the mineral to matrix ratio (M:M). Secondly, another ratio between the absorbance of A872 / A1026 was also calculated to assess the distribution of the carbonate content within the mineral phase, in which 872 cm^-1^ is assigned to ν2 of PO_4_^3-^ vibration.

### Scanning Electron Microscopy (SEM) and Energy Dispersive X-ray Spectroscopy (EDX)

The specimens were treated in a critical-point drier and sputter-coated with gold (JEOL JFC1600; Tokyo, Japan) to enable visualisation of the morphological features using SEM (JSM-IT200 InTouchScope; Tokyo, Japan). Photomicrographs were acquired and examined at magnifications of 500X, 2000X and 3000X. EDX analysis was conducted to quantify the calcium (Ca^2+^), phosphorus (PO_4_), oxygen (O), silver (Ag), iodide (I^-^) and fluoride (F^-^) content within the specimens. Three line and point scans were plotted at different locations on the specimen. The data were processed and reported in terms of total elemental concentration along with graphic representation of line scans.

### X-ray Photoelectron Spectroscopy

An ESCA lab250xi instrument (Thermo Fisher Scientific) equipped with a monochromator Al Kα x-ray irradiation source (power 1486.5 eV) and a charge compensation flood gun was used in the surface investigation. Before XPS measurements were performed, the instrument was calibrated with Au, Cu and Ag metals where the binding energies of Au4f7/2, Cu2p3/2 and Ag3d5/2 were 84.0, 932.6 and 368.2 eV, respectively, after surface cleansing with an Ar-ion gun. All spectra lines were corrected with regard to C1s at 284.6 eV. Each specimen was fixed with a double-sided sticky carbon tape on a sample holder and kept in the preparation chamber overnight under vacuum to remove the adsorbed water from the dentin tubules. After the vacuum reached x10^-7^, the specimen was transferred to the analysis chamber for scanning. For all specimens, the scan area was 850 µm^2^ selected from the dentin root. The scanning and peak-fit process was carried out with Thermo Advantage software (v5.956, Thermo Fisher Scientific). The full-survey spectra were collected over a range of 0-1300 eV at pass energy, dwell time and step size of 150 eV, 50 ms and 1 eV, respectively. The full-survey spectra were collected over a range of 0-1300 eV at a pass energy, dwell time and step size of 150 eV, 50 ms and 1 eV, respectively. While at 20 eV pass energy, 50-ms dwell time and 0.1 eV step size, the narrow scan for C1s, O1s, F1s, N1s, Ca2p, P2p and Ag3d spectra were collected from a different number of scans. The number of scans varied depending on the peak intensity in order to yield smooth and high-resolution peaks.

### Statistical Analysis

Changes in the mean values obtained by microhardness tests (surface, 25-50 µm and 50-100 µm) and nano-indentation analysis (hardness [H] and reduced elastic modulus [E_r_]) of NSSF were compared with the control (no treatment) and other test groups (NaF, SDF). The assumptions of homogeneity of variance and normal distribution were checked using Levene’s and Shapiro-Wilk tests, respectively. One-way ANOVA and Tukey’s multiple comparison were used if variances between materials were equal. The non-parametric Kruskal-Wallis test and Dunnett’s T3 post-hoc multiple comparisons test were used when the variances were unequal. The correlation between M:M and carbonate content was analysed by Spearman’s rank correlation tests. The significance level was set at p < 0.05 (SPSS statistical software, v. 20, IBM).

## Results

**Fig 2:**
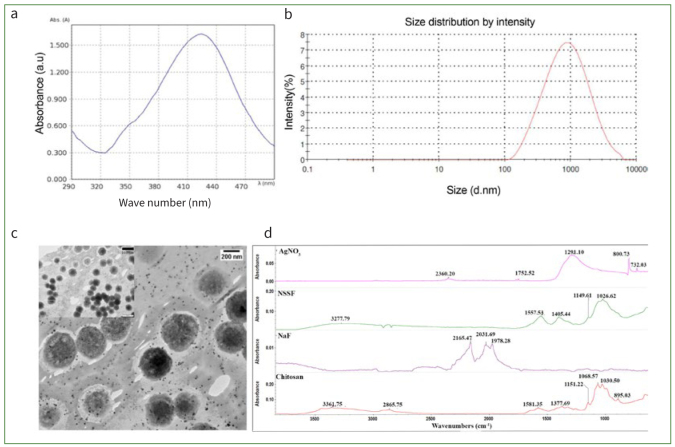
Characterisation of synthesised NSSF by (a) UV-vis spectroscopy with a peak observed at 420 nm; (b) size distribution analysis by Zeta-sizer; (c) TEM of NSSF showing Ag NP and larger fluoride clusters (image scaled at 200 nm) along with a smaller inset image at 500 nm; (d) FTIR spectral data of neat CH, NaF, silver nitrate and prepared NSSF after drying a pellet of the solution on a clean glass slide.

Characterisation of the prepared NSSF is shown in Fig 2. The UV-vis spectral data revealed a characteristic peak at a wavelength of 420 nm, denoted by the presence of Ag nanoparticles (Fig 2a). Particle size analysis revealed an average of 586 nm and a polydispersity index (PdI) of 0.47 (Fig 2b). The TEM showed that the particles were pseudospherical and somewhat monodispersed. The addition of NaF to stabilise the reaction resulted in no aggregation or alteration in the size of the nanoparticles (Fig 2c). FTIR spectra of pure CH showed typical glycosidic linkages, -NH and -OH stretching vibrations, along with amide-I peaks at 1580 cm^-1^. Spectral data acquired from NSSF showed peak shifts to lower wavelengths, with glycosidic fingerprint regions displaying more prominent peaks related to C-O-C bands.

The results from the micro- and nano-mechanical surface observations as well as cross-sectional microhardness and nano-indentation analysis are summarised in Table 2. Compared with the control (no treatment), the other three test groups (NaF, NSSF, and SDF) had statistically significantly higher mean scores for the surface and cross-sectional microhardness (p < 0.05). Among the test groups, SDF showed statistically significantly (p < 0.05) higher mean scores compared with NaF and NSSF for cross-sectional microhardness (Table 2).

**Table 2:** Results of the surface and cross-sectional microhardness (MPa) at depths of 25-50 µm and 50-100 µm below the surface, and nano-indentation analysis represented by hardness (H) (GPa) and reduced elastic modulus (E_r_) (GPa)

	Control (no treatment)		NaF		NSSF		SDF	
	Mean	SD	Mean	SD	Mean	SD	Mean	SD
25-50 µm depth	12.363^a,b,c^	1.842	23.177^a,d^	1.282	20.160^b,e^	5.816	34.790^c,d,e^	3.058
50-100 µm depth	24.843^a,b,c^	1.792	30.693^a,d^	2.350	27.463^b,e^	4.371	39.789^c,d,e^	1.116
Surface	28.118^a,b,c^	3.451	40.479^a^	5.488	38.004^b^	10.233	42.895^c^	3.110
Surface - Er (GPa)	6.47^a^	1.369	4.356^b^	0.734	5.120^c^	1.094	10.237^a,b,c^	1.682
Surface - H (GPa)	0.263	0.109	0.167^a^	0.049	0.158^b^	0.089	0.356^a,b^	0.099

NaF: 5% sodium fluoride; NSSF: nanosilver sodium fluoride; SDF: silver diamine fluoride. Same superscript letters indicate statistically significant differences in the mean values at p < 0.05 (Dunnett’s T3 post-hoc multiple comparisons).

The mean nanohardness (H) and reduced elastic modulus (E_r_) values are shown in Table 2. The E_r_ values for SDF were statistically significantly higher (p < 0.05) compared to the other three groups (control, NaF and NSSF). The H values found using nano-indentation were significantly higher for SDF than for NaF and NSSF (p < 0.05). The mineral to matrix ratio (M:M) and carbonate content within the mineral phase were calculated from the mean peak area and shown in Table 3. No statistically significant correlations were found between the different groups for their M:M and carbonate content (p>0.05). In terms of within-group comparisons, NSSF showed a statistically significant correlation between its M:M and carbonate content (p < 0.05). Other groups did not exhibit similar statistically significant correlations between their M:M and carbonate contents (Table 3).

**Table 3:** Results of the mineral:matrix ratio and carbonate content values calculated from peak area of control, NSSF, NaF and SDF

	Control (No treatment)		NaF		NSSF		SDF	
	Mean	SD	Mean	SD	Mean	SD	Mean	SD
Mineral:matrix ratio	2.944	1.373	2.741	1.444	2.336^a^	0.514	2.371	1.066
Carbonate content	0.022	0.020	0.008	0.003	0.006^a^	0.002	0.010	0.008

NaF: 5% sodium fluoride; NSSF: nanosilver sodium fluoride; SDF: silver diamine fluoride. Same superscript letters indicate statistically significant differences at p < 0.05 using Spearman’s rank correlation.

**Fig 3:**
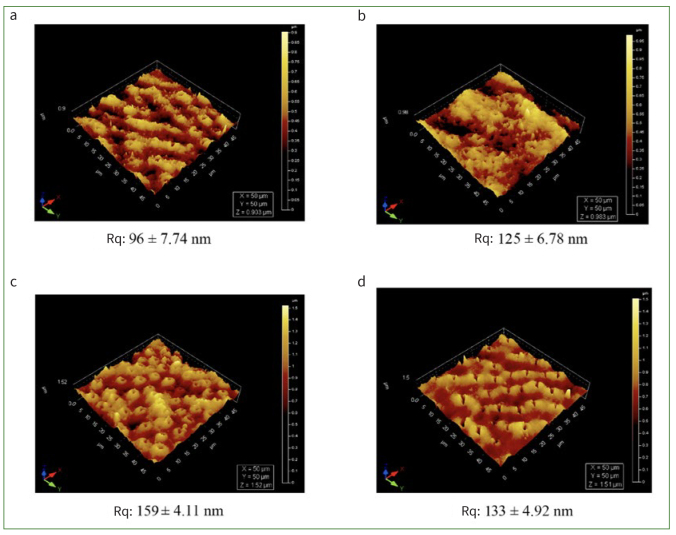
AFM 3D images showing the structural and topographical variations of surface (a) control, (b) NSSF, (c) NaF and (d) SDF showing a 50 x 50 µm area of interest, depicting different morphological observations of dentinal tubules, peritubular and intertubular dentin. Tubular lumen show a different morphology (a) when compared with treated specimens. Rq: the root mean square (RMS) roughness.

Qualitative analysis was performed by AFM to visualise the three-dimensional (3D) topography and morphology of control and experimental specimens after treatment and pH cycling (Figs 3a to 3d). Differences in the surface topography were observed; control (Fig 3a) specimens presented the most irregular and eroded features, indicative of an aggressive effect of demineralisation and pH cycling. The root mean-square roughness of the control (Fig 3a) was 93 nm. Treating the specimens with NSSF, NaF and SDF yielded rougher surfaces of 128, 159 and 132 nm, respectively. The control (Fig 3a) showed enlarged tubules with irregular surface morphology and cross-sectional topography depicting a typical tubular pattern. Samples treated with NSSF, NaF or SDF showed random deposition or precipitation of minerals on the specimen surfaces (Figs 3b to 3d), with no preferential organisation. Also, the lumen of the tubules of SDF specimens exhibited a closed morphology, mimicking occluded tubules.

**Fig 4:**
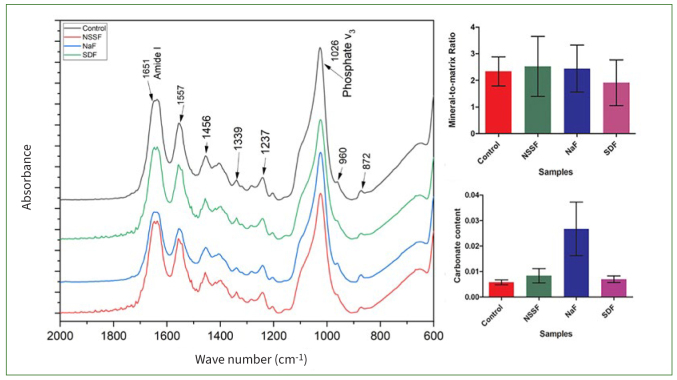
FTIR spectral data of control, NSSF, NaF and SDF. Image shows average statistical spectra depicting the alterations in the phosphate band at 1026 and 872 cm^1^ and amide-I vibration at 1651 cm^1^. Mineral to matrix ratio (M:M) and carbonate content calculated using the peak area tool for control, NSSF, NaF and SDF.

Figure 4 shows mean spectral data (2000^-1^ to 800 cm^-1^) of control, NSSF, NaF and SDF. The absorption bands associated with organic content are seen at 1557 cm^-1^ which is attributed to amide-II peaks. The peak at 1651 cm^-1^ indicates amide I. An amide-III peak is also observed at 1237 cm^-1^. The fingerprint region displayed typical peaks pertaining to hosphate (PO_4_^3-^) band “v” vibrations at 960^-1^ and 1026 cm^-1^. A peak at 1339 cm^-1^ was als notable, and usually assigned to S=O stretching vibration modes of sulphonamide. Also, a B-type carbonate (CO_3_^2-^) substitution peak was visible at 872 cm^-1^.

**Fig 5:**
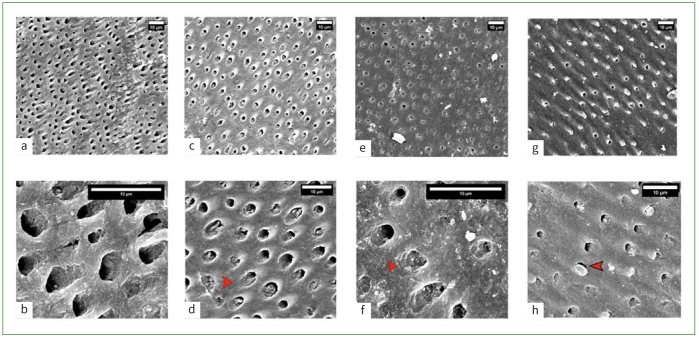
Scanning electron micrographs of (a) control, (c) NSSF, (e) NaF and (g) SDF, all images scaled at 10 µm. Dentinal tubules are clearly visible on control specimens, with treated specimens showing tubules filled with remineralising agents at higher magnification images: (b) control, (d) NSSF, (f) NaF, (h) SDF. Higher magnification images showed that tubules in the control (b) specimens were mineral free, and a clear ring of the peritubular dentin was noted. (d) NSSF-treated specimens showed occluded tubules.

Representative scanning electron micrographs are shown in Fig 5. The demineralisation of dentin did not create a very pronounced erosive ultrastructure (Figs 5a and 5b). There was no mineral precipitation observed on untreated dentin specimens. In contrast, the treated specimens showed occluded tubules (Figs 5c to 5h), and control specimens exhibited open dentinal tubules, which were also visible at higher magnification. NSSF treated specimens showed fewer occluded tubules than did SDF and NaF specimens. Results from the EDX analysis of the control and treated specimens are shown in Table 4. Control specimens had lower levels of calcium (Ca^2+^) than did NSSF and NaF specimens. Higher concentrations of Ag and iodide (I^-^) were noticed for SDF specimens than for NSSF specimens. The intensity of fluoride ions was inconspicuous in the NSSF, NaF and SDF specimens, whereas the control specimens showed obvious peaks of Ca^2+^ and PO_4_ but no obvious signs of F^-^ were observed. The atomic weight data for SDF showed that Ag and I^-^ dominated the surface, with lesser concentrations of Ca^2+^ and PO_4_ (Table 4).

**Table 4:** Energy dispersive x-ray (EDX) spectroscopy analysis of control, NSSF, NaF and SDF specimens

	Ca	P	O	F	Ag	I
Control	42 ± 0.07	23 ± 0.14	33 ± 0.28	N/ A	N/A	N/A
NSSF	44 ± 18.39	17 ± 4.91	37 ± 16	1.12 ± 0.72	N/A	N/A
NaF	43 ± 5.16	21 ± 1.87	22 ± 5.61	2.23 ± 0.84	N/A	N/A
SDF	12 ± 10.06	10 ± 9.05	11 ± 8.89	0.7 ± 0.14	30 ± 13.04	37 ± 12.65

Data shown are the atomic weight % acquired by line scans performed at different regions of the specimens.

**Fig 6:**
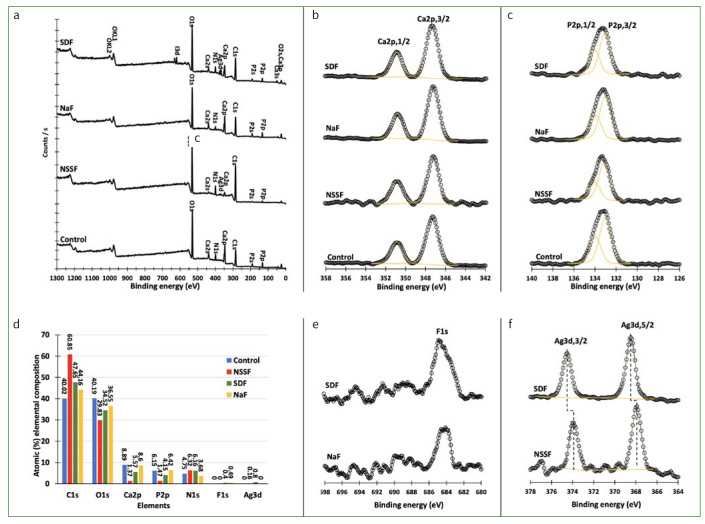
X-ray photoelectron spectroscopy data of (a) surveys of the control, NSSF, NaF and SDF specimens; (b) graph of the calcium content; (c) graph of the phosphorus content; (d) atomic percentage of the elemental composition of control, NSSF, NaF, and SDF; (e) graphs of the fluoride concentration; (f) graphs of the silver concentration from dentin specimens.

The XPS surface examination results of root dentin after demineralisation (control) and remineralisation in NSSF, NaF and SDF are compiled in Fig 6. Figure 6a shows that the surface of the control dentin specimen after demineralisation consisted of carbon, oxygen, nitrogen, Ca^2+^ and P. The same elements were observed on the surface of NaF specimen with variations in the atomic percentage (at%) distribution. Besides these elements, a small amount of Ag was detected after remineralisation of dentin in NSSF, and an appreciable amount of Ag and I^-^ was observed in SDF samples. The high-resolution spectra of Ca2p in the range of 342-358 eV (Fig 6b) revealed no obvious change in the binding energy of Ca2p,3/2 and Ca2p,1/2 after treatment and pH cycling; furthermore, no change in the spectra lines of P2p was observed (Fig 6c). The atomic percentage distribution of the specimens’ surface elemental composition shows that carbon and oxygen were the main components of the dentin surface, followed by Ca^2+^, P, and nitrogen. For instance, C and O are present at about 80.0 at% on the surface of the control specimen, while Ca^2+^, P and N are 8.9, 6.2 and 4.8%, respectively (Fig 6d). It is worth mentioning that F^-^ was detected on the surface of NaF and SDF specimens after 100 scans, while no fluoride was observed on the surface NSSF specimens (Fig 6e). Figure 6f shows the XPS spectra for Ag3d for NSSF and SDF specimens. The spectra lines for Ag3d,5/2 and Ag3d,3/2 at 368.52 and 374.52 eV reveal that Ag is in the metallic state (Ag0) on the surface of SDF specimen, while on the surface of NSSF specimens, the spectra lines measured at 367.94 and 373.88 eV for Ag3d,5/2 and Ag3d,3/2 confirm that silver is present as Ag^+^. The same binding energies for Ag3d,5/2 and Ag3d,3/2 were observed in the as-prepared NSSF.

## Discussion

Based on the results of the current study, the null hypothesis had to be rejected, since there were statistically significant differences in the surface and cross-sectional microhardness of specimens receiving no treatment vs those treated with NSSF, SDF or NaF. However, there were no statistically significant differences between the three groups (NSSF, SDF and NaF); thus, this aspect of the null hypothesis was accepted. The second null hypothesis failed to be rejected as there were no statistically significant differences between the different test groups for the mineral:matrix ratio and carbonate content.

Previous reports conducted on remineralisation of tooth surfaces using NSSF have demonstrated statistically significant effects on the dentin.^8,31,33^ Results of the current investigation indicated that Ag nanoparticles could be successfully synthesised with F^-^ in CH-based solutions, as shown by TEM, UV-vis and FTIR spectroscopy. Earlier studies have reported particle sizes ranging from 2.56 to 3.2 nm, aimed at targeting dentin remineralisation.^18,22^ A study conducted by Widoniak et al^35^ mentioned that particles prepared by hydroquinone as a reducing agent ranged from 500 to 1000 nm. Conversely, those prepared by sodium borohydride were in the range of 300 to 600 nm and displayed irregular shapes.^35^ A similar range was observed in the current study (580 nm). Furthermore, this size and shape could be harnessed by using different polymers, organic salts, temperature and humidity of the chemical reaction.^35^

The dentin specimens after experimental treatment underwent pH cycling using remineralisation and demineralisation solutions. These demineralisation and remineralisation cycles facilitate the acquisition of sufficient data to give researchers the confidence to appropriately plan clinical trials. This step was intended to mimic the in-vivo dynamic alterations in the mineral content and pH levels associated with the natural caries process. It should be noted that while pH cycling was conducted at room temperature, a more realistic outcome could have been achieved with this step being performed at 37°C. Although the degree of particle infiltration in dentin is reliant on the size, there are other factors that might have influenced this procedure in the present study. During the remineralisation step in pH cycling, HA precipitation could occur through a metastable crystalline transition or by epitaxial growth (crystallographic control mechanism).^9,10^ SEM revealed partially occluded tubules in NSSF specimens and fully occluded tubules in SDF and NaF specimens. The EDX analysis indicated that there were nearly undetectable concentrations of Ag and F^-^ in NSSF specimens. This could be due to the low magnification and resolution of the SEM EDX system as reported previously by Sayed et al.^29^ Although some studies have reported that Ag tends to penetrate the tubules, accurate measurements of Ag penetration into demineralised or carious lesions remains challenging as the tubules are arranged differently in different anatomical areas of the tooth.^17^

The principal mineral constituent of dentin is hydroxyapatite. A number of possibilities of different chemical reactions have been hypothesised concerning interactions between HA, Ag^+^ and F^-^ ions.^25^ Sinha et al^30^ demonstrated that use of Ag.based solutions results in an increment in Ca^2+^, PO_4_ and F^-^ ions on caries-affected dentin. This in turn results in the deposition of silver chloride^40^ and formation of fluoridated apatite leading to hardening of the tooth ultrastructure.^30^ The micro- and nano-hardness indentation results reflect this phenomenon. However, some authors have argued that silver phosphate disappears after being immersed in artificial saliva and is replaced by silver chloride or silver thiocyanate.^19^

The microstructural characteristics and chemical composition have a strong correlation with the biomechanical properties of dentin.^9,10^ It has been reported that Ag has an effect on surface and cross-sectional microhardness, nano-indentation, and elastic modulus, but evidence remains inconclusive. Mei et al^19^ reported that the effects of formation nanosilver sodium fluoride upon hardness of a carious lesion is still questionable, as the particles may impair or retard functional remineralisation.^21^ NSSF affects the chemical mineral concentration. These findings agree with the results of the current study. A possible reason for this could be poor mineral precipitation of Ca^2+^ and PO_4_ at the demineralised interface. Therefore, Ag seems to inhibit functional remineralisation and mineral deposition of the dentin matrix.^27^

The representative AFM images revealed diverse profiles of demineralised and treated specimens. Surface roughness was also analysed, which provides an indicator of the surface quality. The tubule openings were also visible, showing different morphological features after demineralisation and treatments with NSSF, NaF and SDF. The technique requires highly polished specimens that could result in removal of some surface features.^36^ The lumen of the dentinal tubules exhibited some narrowing in SDF specimens, indicative of occluded structures.

The main mineral constituent of dentin is composed of phosphate groups and the organic matrix is made up of 90% type-I collagen. The amide-I band is attributed to the peptide group in collagen, and is also correlated with the helical structure of collagen.^39^ The mineral:matrix ratio and carbonate content determination by spectroscopic investigation has been widely used elucidate the distribution of the mineral content. FTIR measures the wavelength and intensity of the absorption of infrared light by a specimen. This IR is energetic enough to excite molecular vibrations to higher energy levels.^5^ FTIR analysis of dentin revealed amide I, II and II bands of collagen and phosphate; carbonate bands of apatite were also visible. They are the main bands for peptide groups in collagen A, which was a common finding; a strong spectral profile of phosphate v3 band at 1026 cm^-1^ was also noted.^5^

The mineralised inorganic content of dentin is composed of HA (phosphate band) and the organic component is made up of type-I collagen (amide-I band). This finding reflects the extent of demineralisation. The M:M clearly suggested the presence and organisation of mineral ions on specimens treated with NSSF, NaF and SDF. Collagen is abundantly available in dentin; its proteolysis has considerable impact on the structural integrity of this hard tissue. This is apparent in dentin affected by carious activity, which can lead to biomechanical and functional loss. Hence, a lower ratio was observed for our control specimens. Minimal changes were noted in the relative intensity of the amide-I band as compared to treated specimens. This observation could be attributed to the loss of interactions between collagen (C=O groups) and Ca^2+^ ions upon demineralisation.^7^

After treatment and pH cycling (demineralisation and remineralisation for 7 days), the carbonate:phosphate ratio showed a progressive incremental change when compared with the untreated samples. It is established that the solubility of PO_4_ groups is enhanced in an acidic environment, whereas carbonates (more specifically in crystalline positions of type B) decrease their solubility at a lower pH. Carbonated apatite (A- or B-type substitution) is a precursor of HA and is commonly revealed in FTIR spectroscopy. Moreover, the carbonate ions replace the phosphate ions within the crystalline ultrastructure of apatite when undergoing demineralisation. As a result, the ratio of the substituted carbonate in the mineral seems to increase as the mineral matures. These alterations could also be indicative of an increment in the crystallinity of the dentin mineral.^10^

The survey spectra confirmed that the surface of dentin specimens is mostly composed of carbon, oxygen and nitrogen due to the presence of collagen, which consists of amino acids and proteins. These elements (C, O and N) covered 97.0% of the surface of NSSF-remineralised specimens with 60.0% coverage with carbon only. The high atomic percentage (at%) of C may originate from the chitosan used in the synthesis of NSSF. The high-resolution spectra of Ca2p and P2p revealed no change in the chemical structure of hydroxyapatite in dentin. While the at% of Ca^2+^ and P were varied, the NSSF specimens demonstrated the lowest at% of Ca^2+^ and P (0.93) compared to 1.45, 1.34 and 1.34 for control, SDF, and NaF specimens respectively. The lower Ca:P ratio for NSSF compared to SDF and NaF specimens could be due to the carbon component, which covered the surface and prevented the remineralisation process. Furthermore, no F^-^ was detected on the NSSF specimen compared to inorganic fluoride measured at about 684 eV with mostly calcium fluoride present on the surface of SDF and NaF specimens.^33^ Moreover, Ag was successfully attached to the surface of SDF and NSSF at an at% of 0.80% and 0.18 at%.

The current study had some limitations. Firstly, it was a laboratory investigation and the demineralisation process might have occurred differently in the clinical setting, leading to different results. Furthermore, this study analysed the short-term in-vitro effects. The long-term effects of the nanoparticles in conjunction with other ions needs to be explored further to confirm the findings of this study. It has also been reported that there are significant differences between the claimed and measured silver and fluoride ion concentration in different 38% SDF products.^37^ Therefore, all in-vitro synthesised nanosilver formulations with fluoride need to be extensively characterised in detail. Moreover, Ag nanoparticles with fluoride can be tailored with respect to particle size, concentration, pH, optical properties, and dielectric constant. These inherent properties could possibly produce a different effect on the demineralised dentin lesions and needs further study in the future.

## Conclusions

The synthesis and characterisation of silver nanoparticles with fluoride using the current method seems to be convenient and feasible for use on demineralised root dentin. Moreover, based on the microhardness, nanohardness and mineral content, NSSF can be considered as an alternative to SDF and NaF as an agent for management of root caries lesions.

## References

[ref1] AlQranei MS, Balhaddad AA, Melo MAS (2021). The burden of root caries: Updated perspectives and advances on management strategies. Gerodontol.

[ref2] Bertassoni LE, Habelitz S, Marshall SJ, Marshall GW (2011). Mechanical recovery of dentin following remineralization in vitro – an indentation study. J Biomech.

[ref3] Cai J, Burrow MF, Manton DJ, Hardiman R, Palamara JEA (2021). Remineralising effects of fluoride varnishes containing calcium phosphate on artificial root caries lesions with adjunctive application of proanthocyanidin. Dent Mater.

[ref4] Cai J, Burrow MF, Manton DJ, Tsuda Y, Sobh EG, Palamara JEA (2019). Effects of silver diamine fluoride/potassium iodide on artificial root caries lesions with adjunctive application of proanthocyanidin. Acta Biomater.

[ref5] de Miranda RR, Silva ACA, Dantas NO, Soares CJ, Novais VR (2019). Chemical analysis of in vivo–irradiated dentine of head and neck cancer patients by ATR-FTIR and Raman spectroscopy. Clin Oral Invest.

[ref6] Detsomboonrat P, Thongmak P, Lertpayab P, Aiemsri W, Sooampon S (2021). Optimal concentration of potassium iodide to reduce the black staining of silver diamine fluoride. J Dent Sci.

[ref7] Di Foggia M, Prati C, Gandolfi MG, Taddei P (2019). An in vitro study on dentin demineralization and remineralization: Collagen rearrangements and influence on the enucleated phase. J Inorg Biochem.

[ref8] dos Santos VE, Filho AV, Ribeiro Targino AG, Pelagio Flores MA, Galembeck A, Caldas AF (2014). A new “silver-bullet” to treat caries in children – nano silver fluoride: A randomised clinical trial. J Dent.

[ref9] Enrich-Essvein T, Benavides-Reyes C, Álvarez-Lloret P, Bolaños-Carmona MV, Rodríguez-Navarro AB, González-López S (2021). Influence of de-remineralization process on chemical, microstructural, and mechanical properties of human and bovine dentin. Clin Oral Invest.

[ref10] Enrich-Essvein T, Rodríguez-Navarro AB, Álvarez-Lloret P, Cifuentes-Jiménez C, Bolaños-Carmona MV, González-López S (2021). Proanthocyanidin-functionalized hydroxyapatite nanoparticles as dentin biomodifier. Dent Mater.

[ref11] Espíndola-Castro LF, Rosenblatt A, Galembeck A, Monteiro GQM (2020). Dentin staining caused by nano-silver fluoride: a comparative study. Oper Dent.

[ref12] Garg S, Sadr A, Chan DCN (2019). Potassium iodide reversal of silver diamine fluoride staining: A case report. Oper dent.

[ref13] Göstemeyer G, Kohls A, Paris S, Schwendicke F (2018). Root caries prevention via sodium fluoride, chlorhexidine and silver diamine fluoride in vitro. Odontol.

[ref14] Hariyani N, Setyowati D, Spencer AJ, Luzzi L, Do LG (2018). Root caries incidence and increment in the population – A systematic review, meta-analysis and meta-regression of longitudinal studies. J Dent.

[ref15] Horst JA, Ellenikiotis H, Committee USCA, Milgrom PM (2016). UCSF protocol for caries arrest using silver diamine fluoride: rationale, indications, and consent. J California Dent Assoc.

[ref16] Lechner B-D, Röper S, Messerschmidt J, Blume A, Magerle R (2015). Monitoring demineralization and subsequent remineralization of human teeth at the dentin – enamel junction with atomic force microscopy. ACS App Mater Inter.

[ref17] Li Y, Liu Y, Psoter WJ, Nguyen OM, Bromage TG, Walters MA (2019). Assessment of the silver penetration and distribution in carious lesions of deciduous teeth treated with silver diamine fluoride. Caries Res.

[ref18] Mei ML, Ito L, Cao Y, Li QL, Lo ECM, Chu CH (2013). Inhibitory effect of silver diamine fluoride on dentine demineralisation and collagen degradation. J Dent.

[ref19] Mei ML, Lo ECM, Chu CH (2018). Arresting dentine caries with silver diamine fluoride: what’s behind it?. J Dent Res.

[ref20] Meyer-Lueckel H, Machiulskiene V, Giacaman RA (2019). How to intervene in the root caries process? Systematic review and meta-analyses. Caries Res.

[ref21] Osorio R, Osorio E, Aguilera FS, Medina-Castillo AL, Toledano M, Toledano-Osorio M (2018). Silver improves collagen structure and stability at demineralized dentin: A dynamic-mechanical and Raman analysis. J Dent.

[ref22] Pentapati KC, Siddiq H, Yeturu SK (2019). Global and regional estimates of the prevalence of root caries – Systematic review and meta-analysis. Saudi Dent J.

[ref23] Qasim SSB, Ali D, Khan AS, Rehman SU, Iqbal A, Baskaradoss JK (2021). Evidence-based bibliometric analysis of research on silver diamine fluoride use in dentistry. BioMed Res Int.

[ref24] Qasim SSB, Ali D, Soliman MS, Zafiropoulos G-G (2021). The effect of chitosan derived silver nanoparticles on mechanical properties, color stability of glass ionomer luting cements. Mater Res Exp.

[ref25] Rosenblatt A, Stamford TCM, Niederman R (2009). Silver diamine fluoride: a caries “silver-fluoride bullet”. J Dent Res.

[ref26] Sadyrin E, Lapitskaya V, Kuznetsova T, Yogina D, Maksyukov S, Aizikovich S (2022). Nanoindentation and atomic force microscopy derived mechanical and microgeometrical properties of tooth root cementum. Micro.

[ref27] Saito T, Toyooka H, Ito S, Crenshaw MA (2003). In vitro study of remineralization of dentin: effects of ions on mineral induction by decalcified dentin matrix. Caries Res.

[ref28] Savas S, Kavrìk F, Kucukyìlmaz E (2016). Evaluation of the remineralization capacity of CPP-ACP containing fluoride varnish by different quantitative methods. J App Oral Sci.

[ref29] Sayed M, Matsui N, Uo M, Nikaido T, Oikawa M, Burrow MF (2019). Morphological and elemental analysis of silver penetration into sound/demineralized dentin after SDF application. Dent Mater.

[ref30] Sinha R, Singh A, Kishor A, Richa S, Kumar R, Kumar A (2011). Evaluation of oral hygiene status in patients with hemorrhagic and ischemic stroke. J Con Dent.

[ref31] Soekanto SA, Fadillah F, Nuraisiya P, Gultom FP, Sarwono AT (2017). The potential of several fluoride-based varnishes as remineralization agents: Morphological studies, dentin surface hardness, and crystallinity tests. Int J App Pharm.

[ref32] Sorkhdini P, Gregory RL, Crystal YO, Tang Q, Lippert F (2020). Effectiveness of in vitro primary coronal caries prevention with silver diamine fluoride – Chemical vs biofilm models. J Dent.

[ref33] Taube F, Ylmén R, Shchukarev A, Nietzsche S, Norén JG (2010). Morphological and chemical characterization of tooth enamel exposed to alkaline agents. J Dent.

[ref34] Teixeira JA, Silva AVCe, Santos Júnior VEd, Melo Júnior PCd, Arnaud M, Lima MG (2018). Effects of a new nano-silver fluoride-containing dentifrice on demineralization of enamel and Streptococcus mutans adhesion and acidogenicity. Int J Dent.

[ref35] Widoniak J, Eiden-Assmann S, Maret G (2005). Silver particles tailoring of shapes and sizes. Colloids Surf A: Physicochem Eng Aspects.

[ref36] Wu Q, Mei ML, Wu X, Shi S, Xu Y, Chu CH (2020). Remineralising effect of 45S5 bioactive glass on artificial caries in dentine. BMC Oral Health.

[ref37] Yan IG, Zheng FM, Gao SS, Duangthip D, Lo ECM, Chu CH (2022). Ion concentration of silver diamine fluoride solutions. Int Dent J.

[ref38] Yin IX, Zhao IS, Mei ML, Lo ECM, Tang J, Li Q, Yu OY, Zhao IS, Mei ML, Lo ECM, Chu CH (2018). Synthesis and characterization of fluoridated silver nanoparticles and their potential as a non-staining 8. J Dent.

[ref39] Zhao IS, Gao SS, Hiraishi N, Burrow MF, Duangthip D, Mei ML (2018). Mechanisms of silver diamine fluoride on arresting caries: a literature review. Int Dent J.

[ref40] Zhao IS, Yin IX, Mei ML, Lo ECM, Tang J, Li Q (2020). Remineralising dentine caries using sodium fluoride with silver nanoparticles: an in vitro study. Int J Nanomed.

